# Editorial: Spotlight on the Background Actors - Physiology and Pathophysiology of Supporting, Accessory and Less Common Cell Types in the Gastrointestinal Tract

**DOI:** 10.3389/fphys.2020.00766

**Published:** 2020-07-21

**Authors:** Pawel E. Ferdek, Monika A. Jakubowska, Wei Huang, Ole H. Petersen

**Affiliations:** ^1^Faculty of Biochemistry, Biophysics and Biotechnology, Jagiellonian University, Krakow, Poland; ^2^Malopolska Centre of Biotechnology, Jagiellonian University, Krakow, Poland; ^3^Department of Integrated Traditional Chinese Medicine and Western Medicine, Sichuan Provincial Pancreatitis Centre and West China-Liverpool Biomedical Research Centre, West China Hospital, Sichuan University, Chengdu, China; ^4^School of Biosciences, Cardiff University, Cardiff, United Kingdom

**Keywords:** gastrointestinal tract (GI tract), Paneth cells, intestinal epithelial cells, bitter taste receptor, parietal cells, pancreatic ductal cells, pancreatic stellate cells (PSCs), tuft cells

The gastrointestinal (GI) tract is an entire system of different organs and tissues, which not only forms a continuous passageway between the mouth and the anus, but also includes the organs that aid food intake and digestion such as the pancreas, liver, gallbladder, or tongue. The primary role of the GI tract is ingestion of food, followed by breakdown and absorption of nutrients as well as removal of the remaining waste. Additionally, the GI tract serves as part of the immune system and maintains the microbial homeostasis by hosting gut microflora and preventing the expansion of potentially harmful bacteria. Given the magnitude and complexity of its roles, it is not surprising that the GI tract comprises vast spectrum of cell types of distinct morphology and specialized functions. However, not all of those cells receive the same amount of scientific attention. Owing to their prevalence or obvious physiological functions, cells such as hepatocytes, enterocytes, or pancreatic acinar cells have been subjects of a large number of studies in the past decades. Many other cell types of lower abundance or less clear physiological roles have not been perceived as equally attractive research targets and as a result have not been extensively investigated or characterized.

As the name of this Research Topic suggests we intended to put in the spotlight somewhat overlooked “background actors” of the GI tract. Although nine articles in this collection might at first seem very diverse in terms of the subjects they touch on, they share a focus on accessory, supporting or less common cell types found in the intestine, stomach, or pancreas. These articles explore physiological roles pertaining to these cells, describe new methodology or provide a concise review of the current knowledge.

An interesting example of an intestinal cell type are Paneth cells. These cells are known for their role in the innate immunity as they secrete antimicrobial peptides. Singh et al. review the literature on metaplastic Paneth cells in order to understand their etiology and the role they play in intestinal metaplasia. The authors conclude that the presence of metaplastic Paneth cells at extra-intestinal mucosal sites could be linked to a protective response induced by the altered microbiome. A study by Burgueño et al. also touches on the subject of gut microbiota. The authors not only describe methodology for measuring epithelial production of H_2_O_2_ but also demonstrate very interesting evidence that intestinal epithelial cells (IECs) generate and release H_2_O_2_ in response to chronic inflammation or as a result of microbial imbalance. This underlies the pivotal role IECs play in the innate defense mechanisms and in maintaining the intestinal homeostasis.

Moving onto the stomach, populations of cells in the gastric epithelium that express a bitter taste receptor Tas2r126 have been identified by Widmayer et al.. Although their role is not yet entirely clear, the authors speculate that these cells may be part of a “surveillance system” that could detect certain constituents of the luminal content and, by conveying this information to the gastric effectors, control appropriate responses of the stomach to the chemical composition of the luminal content. Further, Baratta et al. provide a review of research methodology used in studies on the parietal cells, an epithelial cell type in the stomach whose main function is secretion of hydrochloric acid. The article describes parietal cell isolation and culture as well as discusses the (patho)physiology of these elusive cells and highlights the major research milestones.

Pancreatic cell types have received relatively a lot coverage in this Themed Issue. Gál et al. describe a method of acute isolation of the pancreas that is particularly useful in studying pancreatic ducts and ductal cells. The method is based on injection of low-melting-point agarose into the pancreas via the common bile duct and it allows not only for morphological characterization of these cells but also for functional investigation. A very interesting study comes from DelGiorno et al., who show that chronic pancreatitis (CP) and acinar-to-ductal metaplasia trigger formation of tuft cells in the mouse pancreas and that this process appears to be affected by the mouse genetical background.

Finally, the physiology of pancreatic stellate cells (PSCs), whose activation is a hallmark of pancreatic disorders, has been highlighted by articles from three different groups. Kuntze et al. have investigated the function of mechanosensitive ion channel Piezo1 in these cells and draw conclusions about its role in the pathogenesis of pancreatic ductal adenocarcinoma (PDAC). Stimulation of this channel induces Ca^2+^ influx and is associated with alterations in the cytoskeletal architecture. According to the repot, Piezo1 is sensitive to extra- and intracellular acidification, which may downregulate its excessive activation in response to the mechanical stress exerted by the PDAC stroma and thus protect PSCs against Ca^2+^ overload and cell death. Hu et al. present the role of Yes-associated protein 1 (YAP) in the regulation of PSC activation, proliferation, and fibroinflammatory responses during CP and PDAC progression. The authors also highlight the crosstalk between the YAP, TGF-β, and PDGF signaling pathways, all of which regulate PSC activation and growth. We can learn even more about signaling pathways in PSCs from the article by Kusiak et al., who set the focus of their brief review on the Hippo, Wnt pathways, Ca^2+^ signaling as well as mechanosensing, highlighting the role of all the above in (patho)physiology of the pancreas.

## Author Contributions

All authors listed have made a substantial, direct and intellectual contribution to the work, and approved it for publication.

## Conflict of Interest

The authors declare that the research was conducted in the absence of any commercial or financial relationships that could be construed as a potential conflict of interest.

